# Supplementary material for: Mechanism of histone H2B monoubiquitination by Bre1
Source: bioRxiv. 2023 Aug 28:2023.03.27.534461. Originally published 2023 Mar 29. Preprint. [Version 2] doi: 10.1101/2023.03.27.534461 (PMC10081246; doi:10.1101/2023.03.27.534461)
Supplement: Supplement 1 [file NIHPP2023.03.27.534461v2-supplement-1.pdf]

bioRxiv preprint doi: https://doi.org/10.1101/2023.03.27.534461; this version posted August 28, 2023. The copyright holder for this preprint (which was not certified by peer review) is the author/funder, who has granted bioRxiv a license to display the preprint in perpetuity. It is made available under aCC-BY 4.0 International license.

Table S1. Published effects of Bre1 mutants and LANA peptide competition assay

Mutation/expt	Description	Effect	Reference
Bre1-L650A	Bre1 RING domain contact with Rad6	Defect in ubiquitin discharge from Rad6; Defect in H2B ubiquitination	Gallego et al, PNAS, 2016
Bre1-R675D	Bre1 RING domain contact with Rad6	Defect in ubiquitin discharge from Rad6; Defect in H2B ubiquitination	Gallego et al, PNAS, 2016
Bre1-K682D	Bre1 RING domain contact with Rad6	Defect in H2B ubiquitination	Gallego et al, PNAS, 2016
Bre1-R679D	Arginine anchor in nucleosome acidic patch (Bre1-A); DNA binding (Bre1-B)	Defect in nucleosome binding and H2B ubiquitination	Gallego et al, PNAS, 2016
Bre1-R681D	Arginine anchor in nucleosome acidic patch (Bre1-A); DNA binding (Bre1-B)	Defect in nucleosome binding and H2B ubiquitination	Gallego et al, PNAS, 2016
Bre1-R675D/R679D	Contact with Rad6/ Arginine anchor in nucleosome acidic patch; DNA binding	Defect in nucleosome binding and H2B ubiquitination; Decreased H2B-Ub in yeast; Yeast growth defect	Turco et al JBC 2015
Bre1-R681D/R682D	Arginine anchor in nucleosome acidic patch; DNA binding/Contact with Rad6	Defect in nucleosome binding and H2B ubiquitination; Decreased H2B-Ub in yeast; Yeast growth defect	Turco et al JBC 2015
Competition with LANA peptide	Binds acidic patch; standard test for acidic patch-binding proteins	Disrupts Bre1 binding and H2B ubiquitination	Gallego et al, PNAS, 2016

bioRxiv preprint doi: https://doi.org/10.1101/2023.03.27.534461; this version posted August 28, 2023. The copyright holder for this preprint (which was not certified by peer review) is the author/funder, who has granted bioRxiv a license to display the preprint in perpetuity. It is made available under aCC-BY 4.0 International license.

Table S2. RNF20 mutations located in RING domain and its preceded α helix.

Study of Origin	Cancer Type	Protein Change	Mutation Type
Lung Squamous Cell Carcinoma (TCGA, PanCancer Atlas)	Non-Small Cell Lung Cancer	R949L	Missense_Mutation
Lung Squamous Cell Carcinoma (TCGA, PanCancer Atlas)	Non-Small Cell Lung Cancer	Q954*	Nonsense_Mutation
Uterine Corpus Endometrial Carcinoma (TCGA, PanCancer Atlas)	Endometrial Cancer	R971H	Missense_Mutation
Uterine Corpus Endometrial Carcinoma (TCGA, PanCancer Atlas)	Endometrial Cancer	D915Y	Missense_Mutation
Uterine Corpus Endometrial Carcinoma (TCGA, PanCancer Atlas)	Endometrial Cancer	K929N	Missense_Mutation
Uterine Corpus Endometrial Carcinoma (TCGA, PanCancer Atlas)	Endometrial Cancer	E907A	Missense_Mutation
Uterine Corpus Endometrial Carcinoma (TCGA, PanCancer Atlas)	Endometrial Cancer	L909M	Missense_Mutation
Lung Adenocarcinoma (TCGA, PanCancer Atlas)	Non-Small Cell Lung Cancer	C924F	Missense_Mutation
Skin Cutaneous Melanoma (TCGA, PanCancer Atlas)	Melanoma	H970Y	Missense_Mutation
Stomach Adenocarcinoma (TCGA, PanCancer Atlas)	Esophagogastric Cancer	P923L	Missense_Mutation
Stomach Adenocarcinoma (TCGA, PanCancer Atlas)	Esophagogastric Cancer	R955H	Missense_Mutation
Colorectal Adenocarcinoma (TCGA, PanCancer Atlas)	Colorectal Cancer	R949H	Missense_Mutation
Head and Neck Squamous Cell Carcinoma (TCGA, PanCancer Atlas)	Head and Neck Cancer	P923L	Missense_Mutation
Cervical Squamous Cell Carcinoma (TCGA, PanCancer Atlas)	Cervical Cancer	R949C	Missense_Mutation

bioRxiv preprint doi: https://doi.org/10.1101/2023.03.27.534461; this version posted August 28, 2023. The copyright holder for this preprint (which was not certified by peer review) is the author/funder, who has granted bioRxiv a license to display the preprint in perpetuity. It is made available under aCC-BY 4.0 International license.

Table S3. RNF40 mutations located in RING domain and its preceded α helix.

Study of Origin	Cancer Type	Protein Change	Mutation Type
Bladder Urothelial Carcinoma (TCGA, PanCancer Atlas)	Bladder Cancer	X944_splice	Splice_Site
Uterine Corpus Endometrial Carcinoma (TCGA, PanCancer Atlas)	Endometrial Cancer	R954C	Missense_Mutation
Uterine Corpus Endometrial Carcinoma (TCGA, PanCancer Atlas)	Endometrial Cancer	R954C	Missense_Mutation
Uterine Corpus Endometrial Carcinoma (TCGA, PanCancer Atlas)	Endometrial Cancer	R973W	Missense_Mutation
Uterine Corpus Endometrial Carcinoma (TCGA, PanCancer Atlas)	Endometrial Cancer	E937D	Missense_Mutation
Uterine Corpus Endometrial Carcinoma (TCGA, PanCancer Atlas)	Endometrial Cancer	A944T	Missense_Mutation
Uterine Corpus Endometrial Carcinoma (TCGA, PanCancer Atlas)	Endometrial Cancer	X944_splice	Splice_Region
Uterine Corpus Endometrial Carcinoma (TCGA, PanCancer Atlas)	Endometrial Cancer	A944V	Missense_Mutation
Uterine Corpus Endometrial Carcinoma (TCGA, PanCancer Atlas)	Endometrial Cancer	F967L	Missense_Mutation
Uterine Corpus Endometrial Carcinoma (TCGA, PanCancer Atlas)	Endometrial Cancer	R997H	Missense_Mutation
Stomach Adenocarcinoma (TCGA, PanCancer Atlas)	Esophagogastric Cancer	R973W	Missense_Mutation
Colorectal Adenocarcinoma (TCGA, PanCancer Atlas)	Colorectal Cancer	V966I	Missense_Mutation
Colorectal Adenocarcinoma (TCGA, PanCancer Atlas)	Colorectal Cancer	R973Q	Missense_Mutation
